# Time stamped list mode data from gamma-ray spectroscopic measurements on 47 nuclear fuel assemblies performed at Clab, Sweden, September 2016 through March 2019

**DOI:** 10.1016/j.dib.2020.106039

**Published:** 2020-07-19

**Authors:** Peter Jansson, Martin Bengtsson, Ulrika Bäckström, Sophie Grape, Erik Branger, Anders Sjöland

**Affiliations:** a Dept. of Physics and Astronomy, Uppsala University, Box 516, SE-75120, Uppsala, Sweden; b Swedish Nuclear Fuel and Waste Management Company, Sweden

**Keywords:** Spent nuclear fuel, Gamma, Axial scanning, List mode, Spectroscopy, HPGE, Radiation, Cs-137

## Abstract

Using a high-purity Germanium gamma-ray energy spectroscopic detector system, time-stamped list-mode data sets were acquired during axial scanning of 19 boiling water reactor (BWR) and 28 pressurized water reactor (PWR) type of nuclear fuel assemblies.

The data sets were collected during two measurements campaigns in September 2016 and March 2019 at the Central Interim Storage Facility for Spent Nuclear (Clab) in Sweden. A certified calibration source of ^137^Cs was positioned along the central line of sight between the measured fuel assembly and the detector. Data sets from measurements with only the calibration source and other background sources, i.e. without a nuclear fuel assembly present, are also included.

The list-mode structure of the measured data allows for an axially-resolved as well as energy-spectral resolved intensity of nuclide-specific gamma lines emitted from the spent nuclear fuel. Data presented here can be used e.g. for validation of gamma-ray transport simulation tools or for development of methods to estimate parameters of the spent nuclear fuel based on data from gamma-ray spectroscopy.

**Specifications Table**

**Table:** 

**Subject**	Nuclear Energy and Engineering
**Specific subject area**	High-level radioactive waste management
**Type of data**	Table
**How data were acquired**	Measurements were performed using a high-purity Germanium (HPGe) detector of model GX4018 from Canberra Industries with a relative efficiency of 40%, equipped with cryostat model CP5-Plus-SL and preamplifier model 2002CSL. Time-stamped list-mode data were acquired from the detector using Lynx from Canberra Industries. All data were acquired while a certified ^137^Cs calibration source was positioned along the line of sight of the detector. The ^137^Cs calibration source number AH-6333 of type CDR8252 was certified by Eckert and Ziegler Nuclitec Gmbh, Germany, to have an activity of 3.73 MBq on 1 June 2016 at 12:00 UTC, with a three percent (two-sigma) relative uncertainty.
**Data format**	Raw
**Parameters for data collection**	Trapezoidal shaping of detector pulses from the detector was performed by the Lynx device using a rise time of 2.8 µs and a flat top of 0.6 µs.
**Description of data collection**	Data were collected by saving list-mode detector data during acquisitions. Two types of measurements were performed; Measurements with nuclear fuel assemblies present and background measurements without the fuel assemblies. Background measurements were performed in sequences of about 10 min acquisitions. The fuel assemblies were positioned in the water pool and axially moved during acquisition in front of a collimator built into the pool wall, rotated so that one of its four corners points towards the detector. The ^137^Cs calibration source was positioned along the centre line of the collimator slit both during background and fuel measurements. The detector and associated electronics were located in a room adjacent to the pool.
**Data source location**	Institution: Central Interim Storage Facility for Spent Nuclear (Clab) City/Town/Region: Simpevarp/Oskarshamn Country: Sweden
**Data accessibility**	Repository name: Uppsala university library Data identification number: urn:nbn:se:uu:diva-413179 Direct URL to data: http://urn.kb.se/resolve?urn=urn:nbn:se:uu:diva-413179

**Value of the Data**

• Open access to raw gamma spectroscopic data from measurements on spent nuclear fuel enable independent review of analysis performed in the context of high-level radioactive waste management.
• Developers of methodologies for characterizing and safeguarding spent nuclear fuel and developers of gamma-ray transport simulation codes can benefit from getting access to this data.
• The multi-variate structures in the data can be used to research properties of spent nuclear fuel and to design specialized experiments and/or measurement setups that are customized to specific situations e.g., to gain insight into what is required from a measurement system to be used in the context of transport, storage, encapsulation and disposal of spent nuclear fuel.

## 1 Data description

Measurement data is saved in a text format. All text files with measurement data are saved together in a gzip compressed tar archive, available from the linked repository. The text format is structured as a *measurement header*, followed by a sequence of one or more *meta-batches*. Each meta-batch is structured as a sequence of one or more event *data batches*. Each event data batch is structured as an *event batch header*, followed by one or more *event data* lines and an *event batch footer*. Table 1 shows an example of a data file together with a description of its structure.

**Table 1:** Example of measurement data, displaying the structure of the measurement text file. Some lines were removed for brevity, indicated by “/…/”.

Excerpt from an example data file	Description
# Background acquisition start: 2016–09–26 13:54:52 [UTC]	Measurement header row 1.
# Data in column 1, 2: Event real time [us], Event ADC channel	Measurement header row 2.
# 1 meta-batch(es) follows.	Measurement header row 3.
# 4865 event data batch(es) in meta-batch follows.	Meta-batch header.
# Batch begin at live time 0 us	Start of event data batch.
1314,289	Event data line.
9718,596	Event data line.
12781,3486	Event data line.
14999,2721	Event data line.
15443,5608	Event data line.
/…/	/…/
55577,5113	Event data line.
56144,602	Event data line.
58011,1420	Event data line.
58028,300	Event data line.
62746,56	Event data line.
# Batch end with 28 events	End of event data batch.
# Batch begin at live time 65535 us	Start of event data batch.
66255,2642	Event data line.
66368,143	Event data line.
68312,2321	Event data line.
72862,3822	Event data line.
81147,224	Event data line.
/…/	/…/
150524,22	Event data line.
151569,1434	Event data line.
153842,1173	Event data line.
157804,80	Event data line.
162072,557	Event data line.
# Batch end with 45 events	End of event data batch.
/…/	/… (more event batches)…/
/…/	/… (more event batches)…/
# Batch begin at live time 598179839 us	Start of event data batch.
598180393,1152	Event data line.
598184387,231	Event data line.
598187111,6212	Event data line.
598187187,1438	Event data line.
598187365,83	Event data line.
/…/	/…/
598252659,2537	Event data line.
598253356,452	Event data line.
598258333,2920	Event data line.
598267965,227	Event data line.
598278012,1019	Event data line.
# Batch end with 38 events	End of event data batch.
	/… (next meta-batch) …/

The measurement header contains three rows of information; 1) The acquisition start date and time in UTC. 2) The structure of the event data lines in the later event data batches. 3) The number of meta-event data batches.

The rest of the files contains one or more meta-batches where each meta-batch contains one or more event data batches. Each meta-batch begins with one row of information specifying how many event data batches it contains. All event data batches in the meta-batch follows after the first row of meta-batch information.

Each event data batch begins with a row of the information specifying the live time of the data acquisition system when the batch begins. Then follows the actual list mode data for all events in the event data batch. Each event data row contains two comma-separated integer values; The real time since the start of acquisition and pulse height of the event. The last row of each event data batch specify how many events that were contained in the batch, i.e. how many event data rows already printed before the last row of the event.

All rows of meta-information are prefixed with a hashtag character (‘#’) to indicate that they are not event data rows.

Sequential background measurements are grouped into one text file with each group following the above structure. The background measurements’ file name begins with “background” and contain the start time of the first acquisition contained in the file.

Data from each axial measurement scan of a fuel assembly is saved separately in a text file, structured as described above. The name of the file for fuel assembly measurements begins with the identification of fuel assembly, followed by the acquisition start date and time and ends with information on which corner of the assembly that was measured.

Table 2 displays parameters of the measured fuel assemblies. Table 3 displays information about the background measurements performed. Table 4 displays information about all fuel assembly measurements performed, including acquisition start date and time for each measurement and an association to the fuel assemblies in Table 2 and background in Table 3. Measurements of fuel assemblies used the same lead and copper attenuation plates as those used in the associated background measurement.

**Table 2:** Parameters of measured fuel assemblies. The use of the terms “FuelType 1″, “Fuel Type 2″, …, indicate if the fuel assembly was constructed by the same or different commercial vendors. BU = Burnup, IE = Initial enrichment of ^235^U (weight percent in U). Loading and discharge date refers to the dates when the fuel assembly was loaded and discharged from the reactor. The column “# Cycles” displays the number of irradiation cycles during reactor operation. (*) indicates that the information was unknown. (**) indicates that the fuel assembly was reconstructed after discharge from the reactor. Fuel assemblies identified by BWRnn, PWRnn and BTnn are the same as those measured in references [1], [2], [3], respectively.

Id	Fuel type	BU [GWd/tU]	IE [%]	Loading date	Discharge date	# Cycles
BT01	17 × 17 PWR Fuel Type 6	52.68	3.95	1993–09–27	1997–08–21	4
BT02	17 × 17 PWR Fuel Type 1	55.09	3.95	2010–06–30	2014–08–06	4
BT03	17 × 17 PWR Fuel Type 1	50.29	3.95	2005–08–31	2010–06–17	4
BT04	17 × 17 PWR Fuel Type 1	50.76	3.7	2005–08–31	2009–04–30	4
BT05	17 × 17 PWR Fuel Type 1	50.25	3.6	1994–10–09	2005–08–04	4
BWR02	10 × 10 BWR Fuel Type 2	43.76	3.2	1999–06–09	2004–08–17	5
BWR03	10 × 10 BWR Fuel Type 2	44.36	3.396	1996–08–18	2002–08–12	9
BWR05	10 × 10 BWR Fuel Type 1	42.02	3.14	1999–10–28	2006–08–29	7
BWR06	8 × 8 BWR Fuel Type 1	38.15	2.665	1978–08–05	1985–09–12	9
BWR07	10 × 10 BWR Fuel Type 2	41.24	3.144	1999–06–09	2004–08–17	5
BWR10	10 × 10 BWR Fuel Type 1	39.5	3.14	2001–08–29	2006–08–29	5
BWR11	8 × 8 BWR Fuel Type 1	31.53	2.09	1980–11–08	1992–08–22	7
BWR12	10 × 10 BWR Fuel Type 4	33.51	2.958	1997–06–24	2005–06–10	8
BWR13	10 × 10 BWR Fuel Type 4	36.83	2.958	1997–06–24	2005–06–10	8
BWR14	8 × 8 BWR Fuel Type 1	30.49	2.6435	1978–08–05	1985–09–12	(*)
BWR15	8 × 8 BWR Fuel Type 1	29.42	2.09	1980–11–05	1989–08–25	(**)
BWR16	8 × 8 BWR Fuel Type 1	26.82	2.09	1980–11–05	1987–06–11	(**)
BWR17	8 × 8 BWR Fuel Type 1	32.71	2.31	1975–03–01	1986–07–15	(*)
BWR18	8 × 8 BWR Fuel Type 1	21.52	2.09	1980–11–14	1992–08–22	6
BWR19	8 × 8 BWR Fuel Type 1	30.8	2.578	1984–10–17	1989–06–10	5
BWR21	8 × 8 BWR Fuel Type 1	27.67	2.31	1975–03–01	1987–07–01	(*)
BWR22	10 × 10 BWR Fuel Type 4	20.41	2.962	2001–07–14	2005–06–10	5
BWR24	8 × 8 BWR Fuel Type 2	13.32	1.273	1984–10–17	1987–07–10	3
BWR25	8 × 8 BWR Fuel Type 2	9.13	1.273	1984–10–17	1987–07–10	(*)
PWR01	15 × 15 PWR Fuel Type 1	52.63	3.95	2005–05–04	2009–05–28	4
PWR02	15 × 15 PWR Fuel Type 1	49.555	3.95	2005–05–04	2009–05–29	4
PWR04	17 × 17 PWR Fuel Type 2	46.873	3.95	2004–09–26	2008–06–04	4
PWR05	17 × 17 PWR Fuel Type 2	46.866	3.95	2004–09–26	2008–06–02	4
PWR07	17 × 17 PWR Fuel Type 4	44.483	3.95	2003–09–05	2007–06–27	4
PWR08	17 × 17 PWR Fuel Type 5	44.375	3.29	1984–08–20	1988–09–11	4
PWR09	15 × 15 PWR Fuel Type 2	45.846	3.7	2003–06–15	2007–08–01	4
PWR10	17 × 17 PWR Fuel Type 1	43.474	3.7	1994–07–01	1998–06–17	5
PWR11	17 × 17 PWR Fuel Type 1	43.225	3.6	1994–07–01	2000–06–21	5
PWR12	17 × 17 PWR Fuel Type 5	42.969	3.29	1984–08–20	1988–09–11	4
PWR13	15 × 15 PWR Fuel Type 3	40.92	3.2	1982–07–25	1987–04–25	5
PWR14	17 × 17 PWR Fuel Type 1	40.745	3.6	1993–07–08	1997–06–24	4
PWR15	17 × 17 PWR Fuel Type 5	40.473	2.8	1982–04–17	1987–08–27	4
PWR16	17 × 17 PWR Fuel Type 1	40.41	3.6	1993–06–23	1996–06–21	4
PWR17	17 × 17 PWR Fuel Type 1	40.294	3.7	1994–09–22	1999–09–01	3
PWR18	17 × 17 PWR Fuel Type 1	39.756	3.51	1989–07–09	1995–06–09	4
PWR19	15 × 15 PWR Fuel Type 3	35.027	3.2	1980–05–17	1985–05–01	5
PWR20	17 × 17 PWR Fuel Type 5	34.032	3.1	1980–07–04	1986–06–18	4
PWR21	17 × 17 PWR Fuel Type 5	34.019	3.1	1980–07–04	1986–06–18	4
PWR22	17 × 17 PWR Fuel Type 5	31.165	2.8	1982–04–18	1986–08–10	3
PWR23	17 × 17 PWR Fuel Type 1	28.499	3.6	1993–07–07	1996–06–21	2
PWR24	17 × 17 PWR Fuel Type 5	23.151	2.11	1980–07–02	1995–06–09	4
PWR25	17 × 17 PWR Fuel Type 5	19.607	2.11	1980–07–03	1984–05–24	2

**Table 3:** Information about all background data measurements. The note indicate that a certain width of lead and copper plates were positioned between the detector and the collimator in order to reduce low-energy background radiation hitting the detector. The filename column displays the name of the measurement file within the compressed data archive.

Background id	Acquisition start date	Note	Filename
1	2016–09–26		background_start_2016–09–26 13.54.52.txt
2	2016–09–27		background_start_2016–09–27 12.55.55.txt
3	2016–11–08	4 mm Pb + 1 mm Cu	background_start_2016–11–08 15.40.10.txt
4	2016–11–08		background_start_2016–11–08 16.19.30.txt
5	2016–11–08	4 mm Pb + 1 mm Cu	background_start_2016–11–08 16.42.22.txt
6	2016–11–09		background_start_2016–11–09 06.46.14.txt
7	2016–11–09	10 mm Pb + 1 mm Cu	background_start_2016–11–09 18.31.00.txt
8	2019–03–20	10 mm Pb + 1 mm Cu	background_start_2019–03–20 13.52.05.txt
9	2019–03–27	10 mm Pb + 1 mm Cu	background_start_2019–03–27 18.17.06.txt

**Table 4:** Information about all nuclear fuel measurements. The background and nuclear fuel identification corresponds to the identification used in tables 3 and 2, respectively. The corner number indicate which of the four corners of the fuel assembly that was facing the detector during the axial scan. The filename column displays the name of the measurement file within the compressed data archive.

Id	Measurement date	Corner	Background id	Filename
BT01	2019–03–20	45	8	BT01_start_2019–03–20 12.22.50_corner_45.txt
BT01	2019–03–20	135	8	BT01_start_2019–03–20 12.35.49_corner_135.txt
BT01	2019–03–20	45	8	BT01_start_2019–03–20 12.12.21_corner_45.txt
BT01	2019–03–20	315	8	BT01_start_2019–03–20 13.03.24_corner_315.txt
BT02	2019–03–21	45	8	BT02_start_2019–03–21 07.36.33_corner_45.txt
BT02	2019–03–21	225	8	BT02_start_2019–03–21 08.00.17_corner_225.txt
BT02	2019–03–21	315	8	BT02_start_2019–03–21 08.11.28_corner_315.txt
BT03	2019–03–21	45	8	BT03_start_2019–03–21 09.21.46_corner_45.txt
BT03	2019–03–21	135	8	BT03_start_2019–03–21 09.33.51_corner_135.txt
BT03	2019–03–21	225	8	BT03_start_2019–03–21 09.44.28_corner_225.txt
BT03	2019–03–21	315	8	BT03_start_2019–03–21 09.55.09_corner_315.txt
BT04	2019–03–21	135	8	BT04_start_2019–03–21 11.19.42_corner_135.txt
BT04	2019–03–21	225	8	BT04_start_2019–03–21 11.32.02_corner_225.txt
BT04	2019–03–21	315	8	BT04_start_2019–03–21 11.43.11_corner_315.txt
BT05	2019–03–21	45	8	BT05_start_2019–03–21 12.43.32_corner_45.txt
BT05	2019–03–21	135	8	BT05_start_2019–03–21 12.56.19_corner_135.txt
BT05	2019–03–21	225	8	BT05_start_2019–03–21 13.07.13_corner_225.txt
BT05	2019–03–21	315	8	BT05_start_2019–03–21 13.18.25_corner_315.txt
BWR02	2019–03–26	45	9	BWR02_start_2019–03–26 11.40.57_corner_45.txt
BWR02	2019–03–26	135	9	BWR02_start_2019–03–26 11.54.13_corner_135.txt
BWR02	2019–03–26	225	9	BWR02_start_2019–03–26 12.04.53_corner_225.txt
BWR02	2019–03–26	315	9	BWR02_start_2019–03–26 12.15.25_corner_315.txt
BWR03	2019–03–27	45	9	BWR03_start_2019–03–27 08.27.44_corner_45.txt
BWR03	2019–03–27	135	9	BWR03_start_2019–03–27 08.38.15_corner_135.txt
BWR03	2019–03–27	225	9	BWR03_start_2019–03–27 08.48.42_corner_225.txt
BWR03	2019–03–27	315	9	BWR03_start_2019–03–27 08.59.38_corner_315.txt
BWR05	2019–03–27	45	9	BWR05_start_2019–03–27 07.21.20_corner_45.txt
BWR05	2019–03–27	135	9	BWR05_start_2019–03–27 07.32.07_corner_135.txt
BWR05	2019–03–27	225	9	BWR05_start_2019–03–27 07.42.41_corner_225.txt
BWR05	2019–03–27	315	9	BWR05_start_2019–03–27 07.53.08_corner_315.txt
BWR06	2019–03–27	45	9	BWR06_start_2019–03–27 13.28.52_corner_45.txt
BWR06	2019–03–27	135	9	BWR06_start_2019–03–27 13.38.57_corner_135.txt
BWR06	2019–03–27	225	9	BWR06_start_2019–03–27 13.49.16_corner_225.txt
BWR06	2019–03–27	315	9	BWR06_start_2019–03–27 13.59.40_corner_315.txt
BWR07	2019–03–26	45	9	BWR07_start_2019–03–26 12.51.14_corner_45.txt
BWR07	2019–03–26	225	9	BWR07_start_2019–03–26 13.13.10_corner_225.txt
BWR07	2019–03–26	315	9	BWR07_start_2019–03–26 13.23.49_corner_315.txt
BWR07	2019–03–26	135	9	BWR07_start_2019–03–26 13.36.16_corner_135.txt
BWR10	2019–03–26	45	9	BWR10_start_2019–03–26 10.07.52_corner_45.txt
BWR10	2019–03–26	135	9	BWR10_start_2019–03–26 10.18.52_corner_135.txt
BWR10	2019–03–26	225	9	BWR10_start_2019–03–26 10.29.43_corner_225.txt
BWR11	2019–03–27	45	9	BWR11_start_2019–03–27 09.35.44_corner_45.txt
BWR11	2019–03–27	135	9	BWR11_start_2019–03–27 09.46.19_corner_135.txt
BWR11	2019–03–27	225	9	BWR11_start_2019–03–27 09.56.52_corner_225.txt
BWR12	2019–03–26	45	9	BWR12_start_2019–03–26 14.15.31_corner_45.txt
BWR12	2019–03–26	135	9	BWR12_start_2019–03–26 14.26.05_corner_135.txt
BWR12	2019–03–27	225	9	BWR12_start_2019–03–27 06.37.48_corner_225.txt
BWR13	2019–03–27	45	9	BWR13_start_2019–03–27 14.33.54_corner_45.txt
BWR13	2019–03–27	135	9	BWR13_start_2019–03–27 14.44.10_corner_135.txt
BWR13	2019–03–27	315	9	BWR13_start_2019–03–27 15.04.34_corner_315.txt
BWR14	2019–03–28	45	9	BWR14_start_2019–03–28 09.06.06_corner_45.txt
BWR14	2019–03–28	135	9	BWR14_start_2019–03–28 09.16.31_corner_135.txt
BWR14	2019–03–28	225	9	BWR14_start_2019–03–28 09.27.04_corner_225.txt
BWR14	2019–03–28	315	9	BWR14_start_2019–03–28 09.37.30_corner_315.txt
BWR15	2019–03–28	45	9	BWR15_start_2019–03–28 10.13.09_corner_45.txt
BWR15	2019–03–28	135	9	BWR15_start_2019–03–28 10.23.15_corner_135.txt
BWR15	2019–03–28	225	9	BWR15_start_2019–03–28 10.33.25_corner_225.txt
BWR15	2019–03–28	315	9	BWR15_start_2019–03–28 10.43.43_corner_315.txt
BWR16	2019–03–27	45	9	BWR16_start_2019–03–27 12.20.47_corner_45.txt
BWR16	2019–03–27	135	9	BWR16_start_2019–03–27 12.31.51_corner_135.txt
BWR16	2019–03–27	225	9	BWR16_start_2019–03–27 12.42.12_corner_225.txt
BWR16	2019–03–27	315	9	BWR16_start_2019–03–27 12.52.22_corner_315.txt
BWR17	2019–03–28	135	9	BWR17_start_2019–03–28 07.06.44_corner_135.txt
BWR17	2019–03–28	225	9	BWR17_start_2019–03–28 07.17.10_corner_225.txt
BWR17	2019–03–28	315	9	BWR17_start_2019–03–28 07.27.37_corner_315.txt
BWR18	2019–03–28	45	9	BWR18_start_2019–03–28 12.34.13_corner_45.txt
BWR18	2019–03–28	135	9	BWR18_start_2019–03–28 12.46.13_corner_135.txt
BWR18	2019–03–28	225	9	BWR18_start_2019–03–28 12.57.49_corner_225.txt
BWR18	2019–03–28	315	9	BWR18_start_2019–03–28 13.09.30_corner_315.txt
BWR19	2019–03–27	45	9	BWR19_start_2019–03–27 11.13.06_corner_45.txt
BWR19	2019–03–27	135	9	BWR19_start_2019–03–27 11.23.46_corner_135.txt
BWR19	2019–03–27	225	9	BWR19_start_2019–03–27 11.34.24_corner_225.txt
BWR19	2019–03–27	315	9	BWR19_start_2019–03–27 11.45.03_corner_315.txt
BWR21	2019–03–27	45	9	BWR21_start_2019–03–27 16.48.19_corner_45.txt
BWR21	2019–03–27	135	9	BWR21_start_2019–03–27 16.58.40_corner_135.txt
BWR21	2019–03–27	225	9	BWR21_start_2019–03–27 17.09.08_corner_225.txt
BWR21	2019–03–27	315	9	BWR21_start_2019–03–27 17.19.36_corner_315.txt
BWR22	2019–03–28	45	9	BWR22_start_2019–03–28 08.02.11_corner_45.txt
BWR22	2019–03–28	135	9	BWR22_start_2019–03–28 08.12.06_corner_135.txt
BWR22	2019–03–28	225	9	BWR22_start_2019–03–28 08.22.21_corner_225.txt
BWR22	2019–03–28	315	9	BWR22_start_2019–03–28 08.32.40_corner_315.txt
BWR24	2019–03–27	45	9	BWR24_start_2019–03–27 15.40.48_corner_45.txt
BWR24	2019–03–27	135	9	BWR24_start_2019–03–27 15.51.41_corner_135.txt
BWR24	2019–03–27	225	9	BWR24_start_2019–03–27 16.02.04_corner_225.txt
BWR24	2019–03–27	315	9	BWR24_start_2019–03–27 16.12.54_corner_315.txt
BWR25	2019–03–28	45	9	BWR25_start_2019–03–28 11.24.23_corner_45.txt
BWR25	2019–03–28	135	9	BWR25_start_2019–03–28 11.36.26_corner_135.txt
BWR25	2019–03–28	225	9	BWR25_start_2019–03–28 11.48.08_corner_225.txt
BWR25	2019–03–28	315	9	BWR25_start_2019–03–28 11.59.49_corner_315.txt
PWR01	2016–11–09	135	6	PWR01_start_2016–11–09 16.12.43_corner_135.txt
PWR01	2016–11–09	225	6	PWR01_start_2016–11–09 16.23.27_corner_225.txt
PWR01	2016–11–09	315	6	PWR01_start_2016–11–09 16.34.46_corner_315.txt
PWR01	2016–11–09	45	5	PWR01_start_2016–11–09 16.46.44_corner_45.txt
PWR01	2016–11–09	135	5	PWR01_start_2016–11–09 16.58.36_corner_135.txt
PWR01	2016–11–09	225	5	PWR01_start_2016–11–09 17.09.19_corner_225.txt
PWR01	2016–11–09	315	5	PWR01_start_2016–11–09 17.20.31_corner_315.txt
PWR01	2016–11–09	45	7	PWR01_start_2016–11–09 17.32.00_corner_45.txt
PWR01	2016–11–09	135	7	PWR01_start_2016–11–09 17.44.09_corner_135.txt
PWR01	2016–11–09	225	7	PWR01_start_2016–11–09 17.54.55_corner_225.txt
PWR01	2016–11–09	315	7	PWR01_start_2016–11–09 18.06.02_corner_315.txt
PWR02	2016–11–09	45	6	PWR02_start_2016–11–09 11.31.29_corner_45.txt
PWR02	2016–11–09	225	6	PWR02_start_2016–11–09 11.55.28_corner_225.txt
PWR02	2016–11–09	315	6	PWR02_start_2016–11–09 12.07.39_corner_315.txt
PWR02	2016–11–09	45	5	PWR02_start_2016–11–09 12.20.17_corner_45.txt
PWR02	2016–11–09	135	5	PWR02_start_2016–11–09 12.31.12_corner_135.txt
PWR02	2016–11–09	225	5	PWR02_start_2016–11–09 12.43.21_corner_225.txt
PWR02	2016–11–09	315	5	PWR02_start_2016–11–09 12.54.16_corner_315.txt
PWR04	2016–09–28	45	2	PWR04_start_2016–09–28 09.04.55_corner_45.txt
PWR04	2016–09–28	135	2	PWR04_start_2016–09–28 09.16.12_corner_135.txt
PWR04	2016–09–28	225	2	PWR04_start_2016–09–28 09.27.23_corner_225.txt
PWR04	2016–09–28	315	2	PWR04_start_2016–09–28 09.38.18_corner_315.txt
PWR05	2016–11–07	45	6	PWR05_start_2016–11–07 15.39.21_corner_45.txt
PWR05	2016–11–07	135	6	PWR05_start_2016–11–07 15.52.12_corner_135.txt
PWR05	2016–11–07	225	6	PWR05_start_2016–11–07 16.05.44_corner_225.txt
PWR05	2016–11–07	315	6	PWR05_start_2016–11–07 16.17.43_corner_315.txt
PWR07	2016–09–28	45	2	PWR07_start_2016–09–28 11.11.57_corner_45.txt
PWR07	2016–09–28	135	2	PWR07_start_2016–09–28 11.25.18_corner_135.txt
PWR07	2016–09–28	225	2	PWR07_start_2016–09–28 11.39.29_corner_225.txt
PWR07	2016–09–28	315	2	PWR07_start_2016–09–28 11.53.18_corner_315.txt
PWR08	2016–11–07	135	6	PWR08_start_2016–11–07 17.14.20_corner_135.txt
PWR08	2016–11–07	225	6	PWR08_start_2016–11–07 17.26.35_corner_225.txt
PWR08	2016–11–07	315	6	PWR08_start_2016–11–07 17.38.54_corner_315.txt
PWR09	2016–11–08	135	6	PWR09_start_2016–11–08 14.33.58_corner_135.txt
PWR09	2016–11–08	225	6	PWR09_start_2016–11–08 14.46.49_corner_225.txt
PWR09	2016–11–08	315	6	PWR09_start_2016–11–08 14.59.07_corner_315.txt
PWR09	2016–11–08	45	5	PWR09_start_2016–11–08 15.17.13_corner_45.txt
PWR09	2016–11–08	135	5	PWR09_start_2016–11–08 15.28.56_corner_135.txt
PWR09	2016–11–08	225	5	PWR09_start_2016–11–08 15.41.05_corner_225.txt
PWR09	2016–11–08	315	5	PWR09_start_2016–11–08 15.53.21_corner_315.txt
PWR10	2016–09–27	45	2	PWR10_start_2016–09–27 06.19.46_corner_45.txt
PWR10	2016–09–27	225	2	PWR10_start_2016–09–27 08.06.12_corner_225.txt
PWR11	2016–09–27	135	2	PWR11_start_2016–09–27 09.34.43_corner_135.txt
PWR11	2016–09–27	315	2	PWR11_start_2016–09–27 09.56.55_corner_315.txt
PWR12	2016–11–08	45	6	PWR12_start_2016–11–08 06.55.40_corner_45.txt
PWR12	2016–11–08	135	6	PWR12_start_2016–11–08 07.08.40_corner_135.txt
PWR12	2016–11–08	225	6	PWR12_start_2016–11–08 07.23.40_corner_225.txt
PWR12	2016–11–08	315	6	PWR12_start_2016–11–08 07.35.44_corner_315.txt
PWR13	2016–11–09	45	6	PWR13_start_2016–11–09 13.57.01_corner_45.txt
PWR13	2016–11–09	135	6	PWR13_start_2016–11–09 14.07.50_corner_135.txt
PWR13	2016–11–09	225	6	PWR13_start_2016–11–09 14.19.42_corner_225.txt
PWR13	2016–11–09	315	6	PWR13_start_2016–11–09 14.30.40_corner_315.txt
PWR14	2016–09–27	45	2	PWR14_start_2016–09–27 11.43.55_corner_45.txt
PWR14	2016–09–27	135	2	PWR14_start_2016–09–27 11.55.25_corner_135.txt
PWR14	2016–09–27	225	2	PWR14_start_2016–09–27 12.06.36_corner_225.txt
PWR14	2016–09–27	315	2	PWR14_start_2016–09–27 12.17.35_corner_315.txt
PWR15	2016–09–29	45	2	PWR15_start_2016–09–29 07.44.30_corner_45.txt
PWR15	2016–09–29	135	2	PWR15_start_2016–09–29 07.56.44_corner_135.txt
PWR15	2016–09–29	225	2	PWR15_start_2016–09–29 08.09.18_corner_225.txt
PWR15	2016–09–29	315	2	PWR15_start_2016–09–29 08.21.45_corner_315.txt
PWR16	2016–09–28	45	2	PWR16_start_2016–09–28 06.26.50_corner_45.txt
PWR16	2016–09–28	225	2	PWR16_start_2016–09–28 06.50.57_corner_225.txt
PWR16	2016–09–28	315	2	PWR16_start_2016–09–28 07.02.24_corner_315.txt
PWR17	2016–11–08	135	6	PWR17_start_2016–11–08 08.56.14_corner_135.txt
PWR17	2016–11–08	225	6	PWR17_start_2016–11–08 09.08.41_corner_225.txt
PWR17	2016–11–08	315	6	PWR17_start_2016–11–08 09.21.02_corner_315.txt
PWR18	2016–09–28	45	2	PWR18_start_2016–09–28 12.33.07_corner_45.txt
PWR18	2016–09–28	135	2	PWR18_start_2016–09–28 12.45.48_corner_135.txt
PWR18	2016–09–28	225	2	PWR18_start_2016–09–28 12.57.41_corner_225.txt
PWR18	2016–09–28	315	2	PWR18_start_2016–09–28 13.09.50_corner_315.txt
PWR19	2016–11–08	45	6	PWR19_start_2016–11–08 12.39.50_corner_45.txt
PWR19	2016–11–08	135	6	PWR19_start_2016–11–08 12.52.35_corner_135.txt
PWR19	2016–11–08	225	6	PWR19_start_2016–11–08 13.10.11_corner_225.txt
PWR19	2016–11–08	315	6	PWR19_start_2016–11–08 13.22.33_corner_315.txt
PWR20	2016–09–29	135	2	PWR20_start_2016–09–29 09.33.57_corner_135.txt
PWR20	2016–09–29	225	2	PWR20_start_2016–09–29 09.46.12_corner_225.txt
PWR20	2016–09–29	315	2	PWR20_start_2016–09–29 09.58.09_corner_315.txt
PWR21	2016–09–29	45	2	PWR21_start_2016–09–29 10.37.42_corner_45.txt
PWR21	2016–09–29	135	2	PWR21_start_2016–09–29 10.49.53_corner_135.txt
PWR21	2016–09–29	315	2	PWR21_start_2016–09–29 11.15.02_corner_315.txt
PWR22	2016–11–08	45	6	PWR22_start_2016–11–08 10.20.13_corner_45.txt
PWR22	2016–11–08	225	6	PWR22_start_2016–11–08 10.45.13_corner_225.txt
PWR22	2016–11–08	315	6	PWR22_start_2016–11–08 10.57.09_corner_315.txt
PWR23	2016–09–28	45	2	PWR23_start_2016–09–28 07.54.19_corner_45.txt
PWR23	2016–09–28	135	2	PWR23_start_2016–09–28 08.05.13_corner_135.txt
PWR23	2016–09–28	225	2	PWR23_start_2016–09–28 08.16.42_corner_225.txt
PWR23	2016–09–28	315	2	PWR23_start_2016–09–28 08.27.43_corner_315.txt
PWR24	2016–09–29	45	2	PWR24_start_2016–09–29 06.07.59_corner_45.txt
PWR24	2016–09–29	135	2	PWR24_start_2016–09–29 06.20.25_corner_135.txt
PWR24	2016–09–29	225	2	PWR24_start_2016–09–29 06.32.37_corner_225.txt
PWR24	2016–09–29	315	2	PWR24_start_2016–09–29 06.44.34_corner_315.txt
PWR25	2016–11–07	45	6	PWR25_start_2016–11–07 13.38.45_corner_45.txt
PWR25	2016–11–07	135	6	PWR25_start_2016–11–07 13.53.48_corner_135.txt
PWR25	2016–11–07	225	6	PWR25_start_2016–11–07 14.06.27_corner_225.txt
PWR25	2016–11–07	315	6	PWR25_start_2016–11–07 14.18.28_corner_315.txt

## 2 Experimental design, materials and methods

The measurement system used for performing the measurements is described in more detail in references [1,2]. In summary, the spent nuclear fuel is placed in a fixture in the pool allowing it to be scanned axially, and rotated along its axis, in front of a conical horizontal collimator slit situated in the pool wall in front of the detector. A certified calibration source of ^137^Cs is placed in a fixed position on the central line of sight in the collimator slit at the edge of the collimator slit at its end pointing towards the pool, way from the detector.

The fuel assembly to be measured was rotated so that one of its four corners faced the collimator and detector and the fuel assembly was then scanned axially up and down in front of the detector. Data was acquired during a time period that began before the up-going fuel assembly was in the field-of-view of the collimator and lasted until it has passed the field-of-view on its way down, i.e. each measurement file for a scan contains data from a fuel assembly going up and then going down in front of the detector. Typically, such an up-down axial scan took about 10 min. This procedure was repeated for other corners of the fuel assembly. Note that the ^137^Cs source was present also during these scans, thus requiring counts from the calibration source to be subtracted from the ^137^Cs peak in the energy spectrum from the fuel assembly.

Background measurements were performed without the fuel placed in its fixture. These measurements allows for quantifying the count rate both from the ^137^Cs source and from other background sources, e.g. naturally occurring radiation from the concrete walls of the measurement room in which the detector was placed. Such background data sets were acquired on several occasions between measurements on fuel assemblies.

In both background and fuel assembly measurements, time-stamped list-mode data from the Lynx device were continuously read out in batches and stored on file during data acquisition.

## Declaration of Competing Interest

The authors declare that they have no known competing financial interests or personal relationships which have, or could be perceived to have, influenced the work reported in this article.

## Appendix Supplementary materials

Image, application 1

## References

[1] Jansson P., Tobin S., Liljenfeldt H., Fugate M., Favalli A., A. Sjöland (2016). Axial and azimuthal gamma scanning of nuclear fuel – implications for spent fuel characterization. J. Nucl. Mater. Manag..

[2] Vaccaro S., Tobin S., Favalli A., Grogan B., Jansson P., Liljenfeldt H., Mozin V., Hu J., Schwalbach P., Sjöland A., Trellue H., D. Vo (2016). PWR and BWR spent fuel assembly gamma spectra measurements. J. Nucl. Instrum. Methods Phys. Res. Sect. A..

[3] Jansson P., Bengtsson M., Bäckström U., Svensson K., Lycksell M., A. Sjöland (2020). Data from calorimetric decay heat measurements of five used PWR 17x17 nuclear fuel assemblies. J. Data in Brief.

